# Observations on the Evolution of the Melanocortin Receptor Gene Family: Distinctive Features of the Melanocortin-2 Receptor

**DOI:** 10.3389/fnins.2013.00028

**Published:** 2013-04-10

**Authors:** Robert M. Dores

**Affiliations:** ^1^Department of Biological Sciences, University of DenverDenver, CO, USA

**Keywords:** melanocortin receptors, ACTH, α-MSH, MRAP, MC2R, MC5R, constructive neutral evolution, evolutionary cell biology

## Abstract

The melanocortin receptors (MCRs) are a gene family in the rhodopsin class of G protein-coupled receptors. Based on the analysis of several metazoan genome databases it appears that the MCRs are only found in chordates. The presence of five genes in the family (i.e., *mc1r, mc2r, mc3r, mc4r, mc5r*) in representatives of the tetrapods indicates that the gene family is the result of two genome duplication events and one local gene duplication event during the evolution of the chordates. The MCRs are activated by melanocortin ligands (i.e., ACTH, α-MSH, β-MSH, γ-MSH, δ-MSH) which are all derived from the polypeptide hormone/neuropeptide precursor, POMC, and as a result the functional evolution of the MCRs is intimately associated with the co-evolution of POMC endocrine and neuronal circuits. This review will consider the origin of the MCRs, and discuss the evolutionary relationship between MC2R, MC5R, and MC4R. In addition, this review will analyze the functional evolution of the *mc2r* gene in light of the co-evolution of the *MRAP* (*Melanocortin-2 Receptor Accessory Protein*) gene family.

## Introduction

An analysis of tetrapod (amphibians, reptiles, birds, and mammals) genomes indicates that the melanocortin receptors (MCRs) are a family of five G protein-coupled receptors (GPCRs) genes (i.e., *mc1r, mc2r, mc3r, mc4r, mc5r*) that have been implicated in the mediation of integument pigmentation, appetite regulation, glucocorticoid synthesis, and exocrine gland secretion (Gantz and Fong, 2003; Cone, 2006). A unifying feature of this gene family is that all of the receptors can be activated by one or more of the melanocortin peptides (i.e., ACTH, α-MSH, β-MSH, γ-MSH; Cone, 2006) with varying degrees of efficacy. The melanocortin ligands are derived from the precursor protein proopiomelanocortin (POMC; Nakanishi et al., 1979), a member of the opioid/orphanin gene family (Dores et al., 2002). As a result, the functional evolution of MCRs co-evolved with the *POMC* gene. However, the functional evolution of at least some of the MCRs is also tied to the co-evolution of two other gene families; the *Melanocortin-2 Receptor Accessory Protein* (*MRAP*) gene family (Metherell et al., 2005; Hinkle and Sebag, 2009; Webb and Clark, 2010; Liang et al., 2011; Vastermark and Schiöth, 2011), and the *AGRP/ASIP* gene family (Vastermark and Schiöth, 2011). The later polypeptides function as antagonists or “inverse agonists” for several MCRs. The evolution of the *AGRP/ASIP* gene family has recently been reviewed (Vastermark and Schiöth, 2011) and will not be discussed in this review. Instead, this review will consider the origins of the MCRs and POMC, the origin of the melanocortin-2 receptor (MC2R) and the melanocortin-5 receptor (MC5R), and the co-evolution of MC2R and MRAP.

## The Phylogenetic Distribution of Melanocortin Receptors and POMC

The MCRs are placed in the A-13 family within the rhodopsin class of GPCRs (Horn et al., 2003; Vassilatis et al., 2003). In terms of origin, the MCR gene family appears to be a relatively “recent” addition as compared to other hormone/neuropeptide-activated GPCR gene families such as the vasopressin/oxytocin receptor gene family (Mohr et al., 1996), the CRH receptor gene family (Denver, 2009), or the GnRH receptor gene family (Kah et al., 2006). A search for MCRs in the genome databases of protostomes has not revealed any orthologous genes in these phyla (Vastermark and Schiöth, 2011). In addition among the deuterostomes, it appears that MCR genes also are not present in the genomes of echinoderms, cephalochordates, or urochordates (Vastermark and Schiöth, 2011). However, the presence of *MCR*-related genes in hagfish, lamprey, cartilaginous fish, teleost, and tetrapod genomes (Vastermark and Schiöth, 2011) provides support for the assumption that the MCRs are a chordate gene family.

Coincidentally, orthologous *POMC* genes have been detected in lamprey, cartilaginous fish, teleost, and tetrapod genomes as well (Dores and Baron, 2011). As a result, the proliferation of the paralogous *mcr*-coding genes and the radiation of the paralogous genes in the opioid/orphanin gene family have been influenced by the genome duplication events which have played a critical role in the proliferation of gene families within the various classes of vertebrates (Ohno et al., 1968; Lundin, 1993; Holland et al., 1994).

## Genome Duplication Events and the Evolution of POMC and Melanocortin Receptors

The chordates can be divided into three major lineages, the protochordates represented today by the arrow worm, Amphioxus, and tunicates (superclass Cephalochordata), vertebrates lacking a true jaw such as the lampreys and hagfishes (superclass Agnatha) and the jawed vertebrates such as the cartilaginous fishes, the ray-finned fishes, lobe-finned fishes, and the tetrapods (superclass Gnathostoma). These three major lineages in chordate evolution emerged sequentially; that is the ancestral protochordate lineages are most ancient and the ancestral gnathostome lineages are most recent (Carroll, 1988). There is general agreement that during the radiation of the ancestral agnathans two genome duplication events occurred in a lineage which ultimately gave rise to the ancestral gnathostomes (Ohno et al., 1968; Lundin, 1993; Holland et al., 1994). As a result where there may have been a single copy of a particular gene in the ancestral protochordates, there was now the potential for four paralogous copies of this gene in the gnathostomes. To add to the proliferation of paralogous members within a gene family the modern ray-finned fishes (teleosts) have undergone an additional genome duplication event (Meyer and Van de Peer, 2005), and there is evidence for local gene duplication events in many gnathostome gene families. It should also be noted that gene loss has occurred in several of the gnathostome gene families.

**Figure 1:**
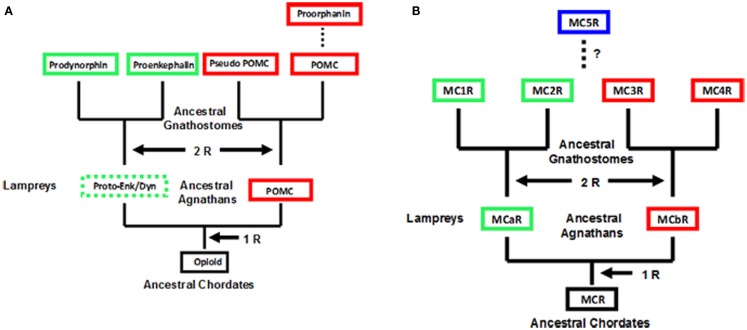
**Scheme for the evolution of opioid/orphanin precursors and melanocortin receptors**. These evolutionary schemes assume that lampreys are 1R organisms. **(A)** Proposed evolution for the opioid/orphanin gene family. The scheme is modified from Dores and Baron (2011). **(B)** Proposed evolution of the melanocortin receptors. This scheme is modified from Baron et al. (2009). The dashed line indicates the origin of MC5R is not resolved. R, refers to the number of genome duplication events; Opioid, ancestral opioid precursor; Proto-Enk/Dyn, hypothetical proto-enkephalin/dynorphin gene; POMC, proopiomelanocortin; MCR, ancestral melanocortin receptor; MCaR, lamprey melanocortin-a receptor; MCbR, lamprey melanocortin-b receptor; MC1R, melanocortin-1 receptor; MC2R, melanocortin-2 receptor; MC3R, melanocortin-3 receptor, MC4R, melanocortin-4 receptor; MC5R, melanocortin-5 receptor.

An operating assumption of the chordate genome duplication process has been that extant agnathans are 1R, where “R” indicates replication of the entire genome, extant gnathostomes are 2R, and the teleosts are 3R. Schemes based on this operating assumption for the opioid/orphanin gene family and the melanocortin receptor gene family is presented in Figure 1. To date neither opioid/orphanin-related genes nor melanocortin receptor-related genes have been detected in the genome of an extant cephalochordate. However, it would be reasonable to propose that the ancestral gene for each gene family emerged in some now extinct protochordate lineage. That said both *POMC*-related genes (Heinig et al., 1995; Takahashi et al., 1995) and *Melanocortin Receptor-*related genes (Haitina et al., 2007), and have been characterized from the lamprey genome.

As shown in Figure 1A, there are two distinct paralogs of the *POMC* gene in the lamprey genome (*POM* and *POC* genes). These genes encode overlapping yet distinct melanocortin and opioid peptides sequences and are expressed in different regions of the lamprey pituitary (Heinig et al., 1995; Takahashi et al., 1995). Enkephalin-like peptides have also been identified in the CNS of the marine lamprey, *Petromyzon marinus* (Dores and Gorbman, 1990) which would suggest that another opioid precursor is present in the lamprey genome. In this scenario, the *Proenkephalin* and *Prodynorphin* genes are the result of the 2 R event, and these genes are found in all the extant groups of gnathostomes; whereas, the *Proorphanin* gene is viewed as the result of a local gene duplication of the *pomc* gene (Sundstrom et al., 2010) which is predicted to have occurred after the 2R event.

The characterization of two melanocortin receptor genes in the lamprey genome (Figure 1B) that are the orthologs of the *MC1R* gene (MCaR) and the *MC4R* gene (MCbR), respectively would be consistent with the assumption that lampreys are 1R organisms (Haitina et al., 2007). In this scenario the second genome duplication event would result in the MC1R, MC2R, MC3R, and MC4R paralogs in the ancestral gnathostomes. At some later point it is assumed the MC5R paralog emerged as a result of a localized gene duplication of one of the other MCR paralogs. The origin of MC5R will be discussed in Section “Origin of MC5R and the Speculations on the Relationship between MC5R and MC4R.” However, there are some aspects of the schemes presented in Figure 1 which challenge the status of the lamprey as a 1R organism. For example, while plausible explanations have been made to explain the presence of three opioid coding genes in the lamprey genome, assuming that lampreys are 1R organisms (Dores et al., 2002), perhaps the status of the extant agnathan genomes needs to be reevaluated.

**Figure 2:**
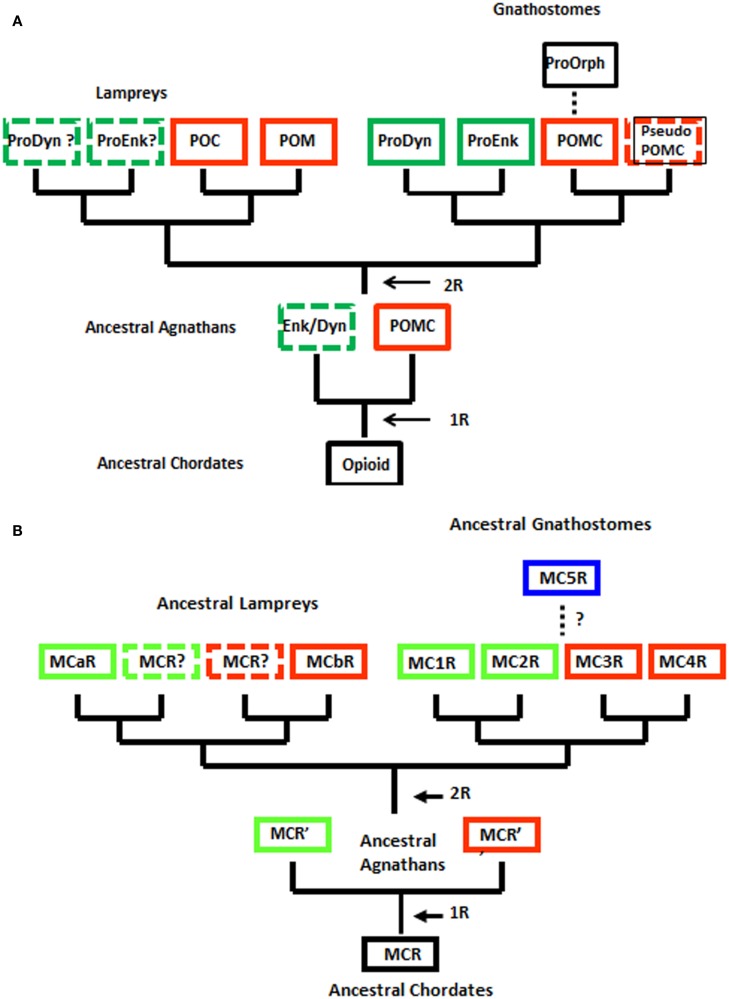
**Alternative schemes for the evolution of opioid/orphanin precursors and melanocortin receptors (MCRs)**. These evolutionary schemes assume that lampreys are 2R organisms. **(A)** Proposed evolution of the opioid/orphanin gene family. **(B)** Proposed evolution of the MCRs. The dashed line indicates the origin of MC5R is not resolved. R, refers to the number of genome duplication events; Opioid, ancestral opioid precursor; Prodyn, prodynorphin; Proenk, proenkephalin, Proorph, proorphanin; Enk/Dyn, hypothetical proto-enkephalin/dynorphin gene; POMC, proopiomelanocortin; POC, proopiocortin; POM, proopiomelanotropin; MCR, ancestral melanocortin receptor; MCaR, lamprey melanocortin-a receptor; MCbR, lamprey melanocortin-b receptor; MC1R, melanocortin-1 receptor; MC2R, melanocortin-2 receptor; MC3R, melanocortin-3 receptor, MC4R, melanocortin-4 receptor; MC5R, melanocortin-5 receptor.

Although the agnathan vertebrates emerged at least 450 million years ago and at their zenith were represented by at least three subclasses and numerous orders (Carroll, 1988), today this superclass has been reduced to two extant subclasses: [Myxini (hagfishes) and Cephalaspidomorphi (lampreys; Nelson, 1994). While the lampreys have been considered a 1R group, recent analyzes of the lamprey genome database (McEwen et al., 2009) have found more members within gene families than would be predicted for a 1R organism. Collectively, these observations have led to the premise that the 2R genome duplication event may have occurred in a group of agnathans that were ancestral to both the lamprey lineage and the ancestral gnathostome lineage (Kuraku et al., 2009; Smith et al., 2013). The ramifications of this hypothesis are reflected in the revised evolutionary trees for opioid/orphanin precursors and for MCRs shown in Figure 2.

When considering the radiation of the opioid/orphanin gene family (Figure 2A), the assumption that the lampreys are 2R organisms provides a more satisfying explanation for the presence of the two *pomc* paralogous genes in the lamprey genome (i.e., *POM* and *POC*). The presence of distinct POM and POC precursor proteins synthesized in the anterior pituitary and intermediate pituitary, respectively, would appear to be the result of the second genome duplication event followed by divergence of the regulatory regions of the *POM* and *POC* genes (Takahashi and Kawauchi, 2006). This scenario also predicts that *Proenkephalin-like* and *Prodynorphin-like* genes may also be present in the lamprey genome. Since the lamprey genome project is only half completed there is likelihood that these other opioid genes may be present. This scenario also is consistent with the current view of the radiation of the opioid/orphanin genes in the gnathostomes (Figure 2A; Sundstrom et al., 2010).

However, regardless of whether the lamprey genome is 1R or 2R, the general organization of POMC has not been radically altered either for the lamprey or the gnathostomes (Vallarino et al., 2012). POMC encodes one copy of a core opioid sequence (YGGF; β-endorphin) and at least one copy of a core melanocortin sequence (HFRW). In the lamprey POC sequence the melanocortin ligand is a highly derived form of ACTH (Heinig et al., 1995). In the lamprey POM sequence there are two melanocortin ligands, melanotropin A and melanotropin B, that correspond to β-MSH and α-MSH, respectively (Takahashi et al., 1995). In the POMC sequences of the cartilaginous fishes there are five melanocortin sequences, ACTH, α-MSH, β-MSH, γ-MSH, and a melanocortin sequence unique to the cartilaginous fishes, δ-MSH (Amemiya et al., 1999). In teleost POMC sequences both the γ-MSH and δ-MSH are absent; whereas, among the tetrapods the γ-MSH sequence is present and there is no equivalent to a δ-MSH sequence (Dores and Lecaude, 2005; Takahashi and Kawauchi, 2006).

It should be noted that the sequence of α-MSH comprises the first 13 amino acids within the ACTH(1–39) sequence. Hence, another feature of the melanocortin network that has been rigorously retained is the differential posttranslational processing of the POMC precursor by the endoproteolytic cleavage enzymes, prohormone convertase 1 (anterior pituitary) and prohormone hormone convertase 2 (intermediate pituitary; Seidah and Chrétien, 1999).

Applying this same scenario to the evolution of the melanocortin receptor genes (Figure 2B), the lamprey genome may contain two additional MCRs. Hence the unique sequence motifs in the MC1R ortholog (MCaR) and the MC4R ortholog (MCbR) may be more derived features than ancestral features. Lamprey MCaR has been expressed in heterologous mammalian cells, and an unexpected observation was that this receptor is selective for ACTH-related analogs, and is much less reactive to MSH-related ligands (Haitina et al., 2007). At present the pharmacology of the lamprey MCbR has not been investigated.

Regardless of whether the lamprey genome is 1R or 2R, the genome of the ancestral gnathostomes should have had at least four paralogous melanocortin receptor genes (i.e., *MC1R*, *MC2R*, *MC3R*, and *MC4R*; Figure 2B), and this feature should be evident in the extant members of this subclass (i.e., the cartilaginous fishes, the bony fishes, and the lobe-finned fishes and tetrapods). To date, all five *MCR* paralogs have been found in the several tetrapod genomes that have been analyzed. However, in teleost genomes some deviations from this scheme have been observed. For example the fugu genomes (*Takifugu rubripes* and *Tetraodon nigroviridis*) lack a *MC3R* gene (Klovins et al., 2004a), and the zebrafish genome has an additional *MC5R* paralog (Ringholm et al., 2002). Finally, with respect to the cartilaginous fishes, while three MCR paralogs (*MC1R, MC2R, MC3r)* have been found in the genome of the holocephalan, *Callorhynchus milii* (Vastermark and Schiöth, 2011), and three MCR paralogs (*MC3R, MC4R*, *MC5R*) have been cloned from the genome of the elasmobranch, *Squalus acanthias*; Ringholm et al., 2003; Klovins et al., 2004b). However, to date all five paralogs have not been characterized from a single cartilaginous fish species. While gene loss could account for the later observation, it should be noted that the *C. milii* genome project has not been completed, and the apparent absence of *mc1r* and *mc2r* from the *S. acanthias* genome may only require a new cloning strategy that takes advantage of the sequence data on the MCR paralogs from the *C. milii* genome project. At this stage it would be reasonable to propose that the ancestral gnathostomes had a minimum of four MCR paralogs (i.e., *MC1R, MC2R, MC3R, MC4R*), which then begs the question of the origin of the *mc5r* gene.

## Origin of MC5R and the Speculations on the Relationship between MC5R and MC4R

When genomes duplicate, paralogous genes will initially be located on distinct homologous chromosomes (Holland et al., 1994). However, it is appreciated that one or both of the paralogs could be subsequently lost, or that non-homologous chromosomes could fuse resulting in two paralogs on the same chromosome. However, the distribution of paralogous genes on distinct chromosomes has been considered a clear indication that a genome duplication event has occurred (Ohno et al., 1968; Lundin, 1993). For example, an analysis of the human, mouse, chicken, fugu (*Takifugu rubripes*), and zebrafish (*Danio rerio*) genomes revealed that the *MC1R* gene, the *MC2R* gene, the *MC3R* gene, and the *MC4R* gene are all located on different chromosomes (Schiöth et al., 2003; Klovins et al., 2004a). In addition, in all six genomes the *MC5R* gene was located on the same chromosome as the *MC2R* in relatively close proximity. These observations provide support for the hypothesis that the *MC5R* gene was the result of a local duplication of the *MC2R* gene (Klovins et al., 2004a). In this scenario ancestral gnathostomes are viewed as having a *MC5R/MC2R* proto-gene, which gave rise to a distinct *mc2r* gene and a distinct *mc5r* gene on the same chromosome as a result of the local gene duplication event (Baron et al., 2009).

Recently these conclusions on the relationship between the *MC2R* and the *MC5R* gene have been called into question (Vastermark and Schiöth, 2011). The issue is that a comparison of the amino acid sequences of MC2R and MC5R indicate that these two MCRs vary considerably in amino acid identity. However, when the amino acid sequences of MC4R and MC5R are compared, these two receptors share a number of identical residues. Furthermore, in a phylogenetic analysis of human and cartilaginous fish MCRs sequences, the MC4R and the MC5R sequences formed a clade (Vastermark and Schiöth, 2011). Based on these observations, Vastermark and Schiöth (2011) predicted that it is more likely that the *MC5R* gene was the result of a local duplication of the *MC4R* gene.

Are these two interpretations for the origin of the *MC5R* gene mutually exclusive? The *MC4R/MC5R* duplication could have occurred at an ancestral gnathostome locus prior to the divergence of the ancestral cartilaginous fishes and the ancestral bony fishes. In this scenario the *MC5R* locus could have moved to the chromosome carrying the *MC2R* locus in a common ancestor prior to the divergence of the ancestral cartilaginous fishes and the ancestral bony fishes. However, when genes duplicate, either as a result of a local duplication event or as a result of a genome duplication event, the new copies of the ancestral gene will accumulate mutations independent of each other. Furthermore based on selection pressures, these independently evolving genes could retain separate functions of the ancestral gene (subfunctionalization) or become adapted for some new function (neofunctionalization; Force et al., 1998).

**Figure 3:**
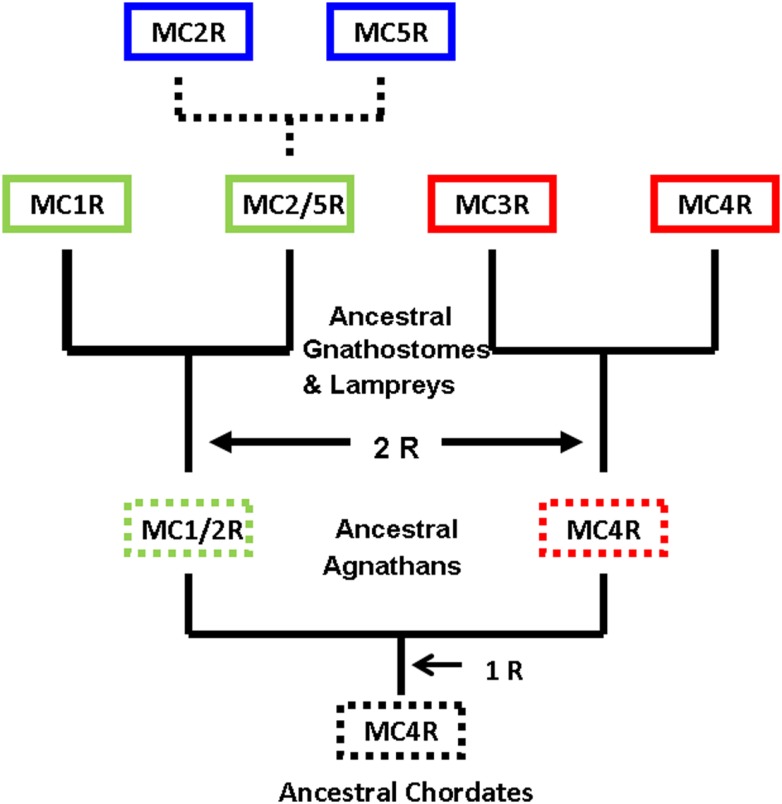
**Revised scheme for the evolution of the melanocortin receptors**. This evolutionary scheme assumes that the melanocortin-4 receptor was the ancestral melanocortin receptor. The dashed box around a receptor indicates that the receptor is predicted but has not been identified. The dashed lines represent the hypothesis that the *MC2R* gene and the *MC5R* gene were the result of a local duplication of the proposed *MC2R/MC5R* gene.

Figure 3 provides an alternative interpretation for the origin of *MC5R* gene that combines the major aspects of the two primary studies (Schiöth et al., 2003; Vastermark and Schiöth, 2011). Figure 3 is based on the assumption that the *MC4R* gene was the ancestral melanocortin gene. A corollary to this assumption is that MCR paralogs would contain a “MC4R” signature; that is, sets of amino acid motifs derived from the proposed ancestral *MC4R* gene. In this scenario a *MC2R/MC5R* gene in the ancestral gnathostomes could have undergone a local gene duplication event. This assumption would be consistent with the synteny studies (Schiöth et al., 2003). Following the duplication event, selection pressures may have favored the *MC5R* duplicate gene maintaining the sequence features found in MC4R, while the *MC2R* duplicate gene apparently accumulated mutations and as a result evolved new functional properties not found in any of the other MCR paralogs.

**Figure A1:**
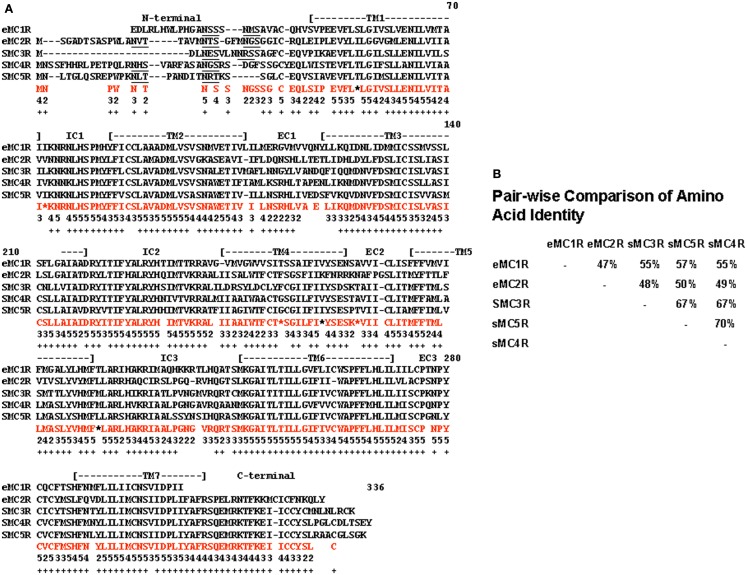
**Alignment of cartilaginous fish melanocortin receptors**. **(A)** The amino acid sequences of the elephant shark (*C. milii*) MC1R (AAVX01456471.1; http://esharkgenome.lmcb.a-star.edu.sg), elephant shark MC2R (accession number: FAA704.1), dogfish (*Squalus acanthias*) MC3R (accession number: AAS66720.1), dogfish MC4R (accession number: AA039833.1), and dogfish MC5R (accession number: AA867890.1) were aligned as described in Liang et al. (2011). In amino acids in red are the consensus amino acids for each position. Consensus was defined as two or more of the same amino acid at that position. Number below each consensus amino acid indicates the number of times that amino acid is present at the position. Positions marked by * there were two pairs of amino acids. These positions were not used in the analysis presented in Figure A2. Positions in which there was no consensus were left blank. The underlined amino acids indicate a N-linked glycosylation site. **(B)** A pair-wise comparison of sequence identity for these receptors is included.

**Figure A2:**
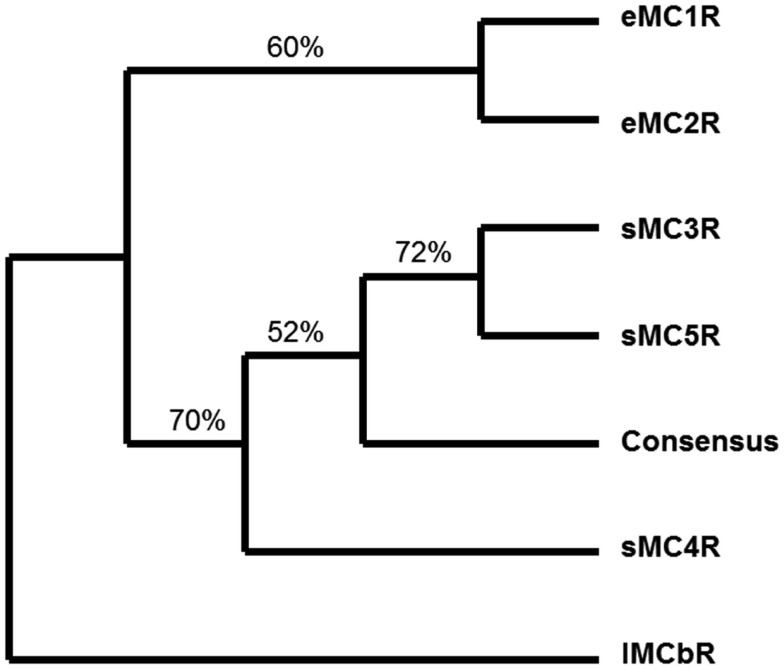
**Maximum parsimony analysis of cartilaginous fish melanocortin receptors**. The sequences presented in Figure A1 including the consensus sequence were analyzed by maximum parsimony using the heuristic program in PAUP. The analysis yield a single tree and the jackknife values for 100 repetitions are included. The outgroup for the rooted tree was the lamprey MCb receptor (accession number: DQ213060) sequence. eMC1R, elephant shark MC1R; eMC2R, elephant shark MC2R; sMC3R, dogfish MC3R; sMC4R, dogfish MC4R; sMC5R, dogfish MC5R.

A quick test of the preceding hypothesis would be to take a set of MCR sequences from the same group of organisms, and identify the consensus sequence common to these receptor. In Figure A1A in Appendix, the amino acid sequences of five cartilaginous fish MCRs were aligned, and common residues that are found at each position are identified in red. The consensus residues at 287 positions were identified. Interesting, 88% of these residues were present in the MC4R sequence. A pair-wise comparison (Figure A1B in Appendix) indicated that the MC4R sequence had the highest sequence identity for the MC5R sequence and the MC3R sequence, respectively. A maximum parsimony analysis of the sequences in Figure A1 in Appendix indicated that the MC4R, the consensus sequence, and the MC3R and MC5R sequences formed a clade (Figure A2 in Appendix). While this correlation analysis is suggestive, an analysis of the hagfish genome may be more useful for testing the validity of the hypothesis presented in Figure 3.

## Evolution of MC2R Ligand Selectivity

As noted in the Introduction, the ligands for the MCRs are the melanocortins, ACTH, α-MSH, β-MSH, γ-MSH (Gantz and Fong, 2003), and for cartilaginous fishes, δ-MSH (Takahashi and Kawauchi, 2006). The sequences of the melanocortins derived from human POMC are presented in Table 1. The proposed origin and the primary sequence variability of vertebrate melanocortins have been reviewed recently (Dores and Baron, 2011). In brief, α-MSH is derived from the first 13 amino acids in the sequence of ACTH via posttranslational processing mechanisms (Eipper and Mains, 1980). It would appear that the γ-MSH and β-MSH are the result of duplications and reinsertions of the α-MSH sequence within the *POMC* gene (Dores et al., 2003); whereas, the δ-MSH sequence appears to be derived from a duplication and reinsertion of the β-MSH sequence in the *POMC* gene of cartilaginous fishes (Amemiya et al., 1999).

**Table 1:** **Human melanocortin ligands**.

ĀCTH	SYSME 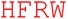 GKPVG 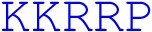 VKVYPNGADDESAEAFPLEF
α-MSH	NAc-SYSME 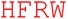 GKPV-NH2
β-MSH	DEGPYRME 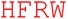 GSPPKD
γ-MSH	KYVMG 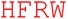 DRF-NH2

*The human melanocortin ligand sequences were derived from the sequence of human POMC (accession # CAG46625.1). The HFRW motif highlighted in red is required for the activation of all melanocortin receptor. The KKRRP motif highlighted in blue is required for the activation of the melanocortin-2 receptor (Schwyzer, 1977)*.

Several studies on mammalian MCRs [reviewed by Gantz and Fong (2003)], as well as a study on bird MCRs (Ling et al., 2004), and studies on teleosts (Ringholm et al., 2002; Klovins et al., 2004a) and cartilaginous fish MCRs (Ringholm et al., 2003; Klovins et al., 2004b; Reinick et al., 2012a) indicate that MC1R, MC3R, MC4R, and MC5R can be activated by ACTH or any of the MSH-sized ligands with varying degrees of efficacy. As indicated in Table 1, all of the melanocortin ligands have the HFRW motif which is required for activation of all MCRs (Schwyzer, 1977; Mountjoy et al., 1992; Gantz and Fong, 2003). From the perspective of the receptors, Pogozheva et al. (2005) identified critical amino acid positions in transmembrane regions 2, 3, 6, and 7 of human MC4R which are required for activation of that receptor by α-MSH. These residues are conserved in other mammalian MCRs (Pogozheva et al., 2005) and have been found in the sequences of MCRs of non-mammalian tetrapods, amphibian MCRs, teleost MCRs, and lamprey MCRs (Baron et al., 2009; Dores, 2009).

That said, none of the MSH-sized ligands in Table 1 can activate either teleost or tetrapod MC2R (Schwyzer, 1977; Mountjoy et al., 1992; Gantz and Fong, 2003; Agulleiro et al., 2010; Liang et al., 2011). Since teleost and tetrapod MC2Rs have retained many of the residues associated with the HFRW binding site in MC1R, MC3R, MC4R, and MC5R, it would appear that the HFRW binding site in teleost and tetrapod MC2Rs is masked in some manner. The apparent key to unmasking the HFRW binding site appears to reside in the KKRRP motif in the sequence of ACTH (Table 1; Schwyzer, 1977; Costa et al., 2004; Liang et al., 2013a). The KKRRP motif is not present in any of the MSH-sized ligands. In addition, either deletions (Schwyzer, 1977) of this motif, or alanine substitutions (Liang et al., 2013a) within this motif will greatly decrease the potency of the ligand. All of these observations point to a KKRRP binding site in teleost and tetrapod MC2Rs, and raise the question of when MC2R orthologs became exclusively selective for ACTH.

**Figure 4:**
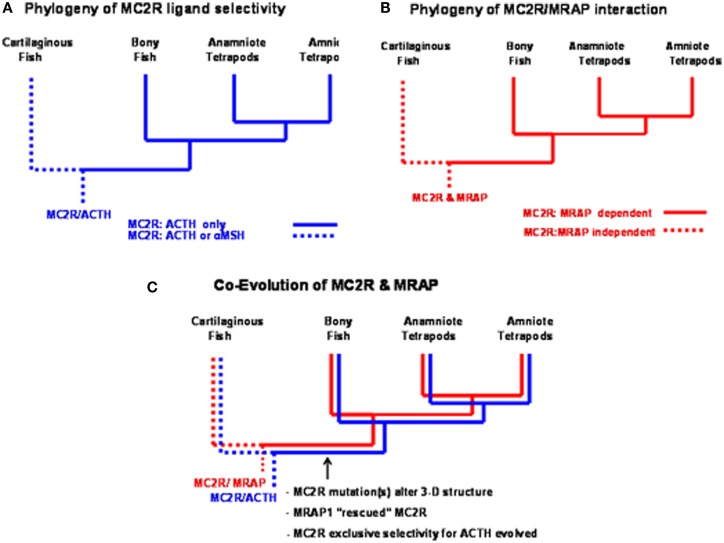
**Functional evolution of the melanocortin-2 receptor**. **(A)** This summary of ligand selectivity of melanocortin-2 receptor orthologs indicates that teleost and tetrapod melanocortin-2 receptors can only be activated by ACTH, but not by any of the MSH-sized melanocortin ligands. The melanocortin-2 receptor ortholog of the cartilaginous fish, *Callorhynchus milii* can be activated by either ACTH or MSH-sized ligands (Reinick et al., 2012b). **(B)** This summary of the melanocortin-2 receptor interaction with MRAP1 indicates that teleost and tetrapod MC2R orthologs are dependent on MRAP1 for trafficking to the plasma membrane and for functional activation at the plasma membrane. The trafficking to the plasma membrane and the functional activation of the MC2R ortholog of the cartilaginous fish, *C. milii* is MRAP1 independent. **(C)** Following the divergence of the cartilaginous fishes and the bony fishes, an interaction developed between MC2R and MRAP1. As mutations occurred in the melanocortin-2 receptor, the receptor was rescued by MRAP1. In extant teleosts and tetrapods, the functional expression of MC2R is dependent on forming a complex with MRAP1.

Studies on a MC2R ortholog in the genome of the holocephalan cartilaginous fish, *Callorhynchus milii*, have provided an opportunity to address the latter question (Reinick et al., 2012b). Expression of the *C. milii MC2R* ortholog in CHO cells indicated that this receptor could be activated by either human ACTH(1–24) or NDP-MSH. In addition, stimulation of *C. milii MC2R* transiently transfected CHO cells with spiny dogfish (*Squalus acanthias*) ACTH(1–25), α-MSH, β-MSH, γ-MSH or δ-MSH yielded dose response curves with varying degrees of efficacy (Reinick et al., 2012b). Although the sample size is small, it is possible that other cartilaginous fish MC2R orthologs have similar ligand selectivity properties. If so, then the summary presented in Figure 4A would indicate a dichotomy in MC2R ligand selectivity between the cartilaginous fishes and the teleosts and tetrapods. In this scenario it is assumed that the MC2R ortholog in the ancestral gnathostomes could also be activated by ACTH or the MSH-related peptides. Hence, the exclusive selectivity for ACTH would appear to have evolved after the divergence of the ancestral cartilaginous fishes and the ancestral bony fishes.

## Co-Evolution of MC2R and MRAP; an Example of Constructive Neutral Evolution

**Figure 5:**
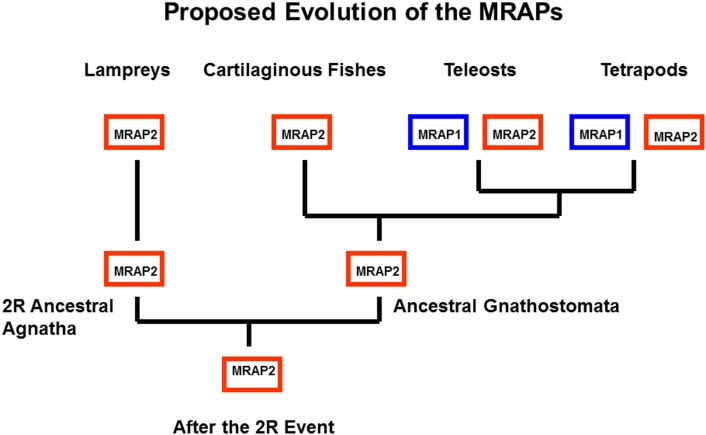
**Proposed Evolution of MRAP1 and MRAP2**. It appears that MRAP2 may be the ancestral paralog in the MRAP gene family. This evolutionary scheme assumes that the *MRAP2* gene was in the genome of the ancestral 2R vertebrates. This gene has been retained in the lamprey genome (Vastermark and Schiöth, 2011). In this scenario only a *MRAP2* gene was present in the genome of the ancestral gnathostomes. Following the divergence of the cartilaginous fishes and the bony fishes, the *MRAP2* gene duplicated in the bony fish lineage, and as a result *MRAP2* and *MRAP1* paralogs are present in the genomes of teleosts and tetrapods.

Another feature of teleost and tetrapod MC1Rs, MC3Rs, MC4Rs, and MC5Rs is that these receptors can be functionally expressed in heterologous mammalian cell lines such has HEK-293 cells, CHO cells, or COS cells (Rachel et al., 2005; Schiöth et al., 2005). These observations are in sharp contrast to teleost and tetrapod MC2Rs which cannot be functionally expressed in those cells lines unless the cells are co-transfected with accessory protein *MRAP1* cDNA (Hinkle and Sebag, 2009; Agulleiro et al., 2010; Webb and Clark, 2010; Liang et al., 2011). Melanocortin-2 Receptor Accessory Protein 1 (MRAP1) is a transmembrane protein with a single transmembrane domain (Metherell et al., 2005). The features of this accessory protein are discussed in another chapter in this book (Clark and Chan, 2013). For the purposes of this review, the salient features of MRAPs include: (a) there are two *MRAP* paralogous genes (*MRAP1* and *MRAP2*) in the vertebrate genome; (b) MRAP1 and MRAP2 form antiparallel homodimers; and (c) MRAP1 is required for the trafficking of MC2R from the ER to the plasma membrane, and for the functional activation of MC2R at the plasma membrane following the binding of ACTH; and (d) MRAP2 can only facilitate the trafficking of MC2R to the plasma membrane, but has a very weak effect on the functional activation of the receptor at the plasma membrane (Hinkle and Sebag, 2009; Webb and Clark, 2010; Gorrigan et al., 2011). In terms of the phylogeny of the *MRAP* genes, it appears that these genes may be restricted to the lamprey genome and the genomes of gnathostomes (Vastermark and Schiöth, 2011). In addition, the two *MRAP* paralogs are not uniformly distributed in these organisms. As summarized in Figure 5, to date only an *MRAP2* gene has been detected in the genome databases for the marine lamprey (*Petromzyon marinus*) and the cartilaginous fish, *Callorhynchus milii*; whereas, *MRAP1* and *MRAP2* genes have been detected in the genomes of several species of teleost fishes, the chicken (*Gallus gallus*) genome, and the genomes of several mammals (Agulleiro et al., 2010; Liang et al., 2011; Vastermark and Schiöth, 2011). It would appear that a duplication of the *MRAP* gene occurred during the radiation of the ancestral bony fishes (Figure 5) resulting in distinct *MRAP1* and *MRAP2* genes. In addition, it is now clear that the functional expression of teleost and tetrapod MC2Rs is dependent on interaction with MRAP1 beginning right after synthesis of MC2R at the rough endoplasmic reticulum (Sebag and Hinkle, 2007).

A *Mc2r* ortholog has been detected in genome of the cartilaginous fish, *C. milli* (Vastermark and Schiöth, 2011). When this *MC2R* ortholog was transiently transfected in CHO cells the receptor could be activated by either ACTH or MSH-sized ligand. Hence, the functional expression of the *C. milli* MC2R ortholog is MRAP1 independent (Reinick et al., 2012b). In addition, the functional expression of the *C. milii* MC2R was not affected, either in a positive or negative manner, following co-expression with either mouse MRAP1, zebrafish MRAP1, or *C. milii* MRAP2. Once again the sample size is small, but as summarized in Figure 4B, the MC2Rs of teleosts and tetrapods are MRAP1 dependent; whereas, the MC2R of at least one species of cartilaginous fish is MRAP independent. This conclusion would suggest that the MC2R ortholog of ancestral gnathostomes was also MRAP1 independent.

Collectively, these observations point to a number of changes and serendipitous events that have occurred during the evolution of the *MC2R* gene. To understand the functional evolution of the *MC2R* gene it may be easiest to start with the current status of this gene in mammals. In all mammals, MC2R serves as the “ACTH” receptor on cells of the adrenal cortex, and is a critical component of the hypothalamus/pituitary/adrenal axis (HPA; Clark and Cammas, 1996). Activation of MC2R results in the synthesis and release of the glucocorticoid, cortisol, a steroid that influences the normal function of many cell types, and facilitates the body’s response to chronic stressors (Engelmann et al., 2004). In humans, mutations in the *MC2R* gene that either effect the trafficking of MC2R from the ER to the plasma membrane or inhibit residues on the receptor responsible for binding ACTH will result in Type 1 Familial Glucocorticoid Deficiency (FGD; Chung et al., 2008). However, the functionality of mammalian MC2Rs is totally dependent on the interaction with MRAPα, one of two splice variants of the human *MRAP1* gene (Metherell et al., 2005). Hence, mutations to critical regions in the human *MRAP1* gene will result in Type 2 Familial Glucocorticoid Deficiency (Metherell et al., 2005). In the absence of a functional MRAP1, *in vitro* experiments indicate that the MC2R is misfolded (Sebag and Hinkle, 2007) and is tagged for degradation by the ER protein quality control system. For either Type 1 or Type 2 FGD, the congenital defect is potentially life threatening if not treated.

Projecting the preceding observations to the functional activation of non-mammalian tetrapod and teleost MC2Rs, *in vitro* experiments have demonstrated that an amphibian MC2R ortholog (Liang et al., 2011) and teleost MC2R orthologs (Klovins et al., 2004a; Agulleiro et al., 2010; Liang et al., 2011) cannot be functionally expressed unless the MC2R ortholog is expressed in cells derived from a mammalian adrenal cell line (that presumably is expressing an endogenous *MRAP1* gene), or in the case of HEK-293 or CHO cells, the *MC2R* ortholog is co-expressed with a *MRAP1* cDNA. Given these observations, it would be reasonable to predict that either functional mutations in the *MC2R* gene, or mutations to the *MRAP1* gene will have a negative effect on the fitness of non-mammalian tetrapods and teleosts.

Hence, the detection of an MRAP1 independent *MC2R* ortholog in the *C. milii* genome that can be activated by either ACTH or MSH-sized ligands would suggest that the *MC2R* gene present in the ancestral gnathostomes after the 2R genome duplication event (Figure 3) was also MRAP independent and perhaps capable of being activated by either ACTH or MSH-sized ligands. This ancestral gene could have been the MC2R/MC5R proto-gene proposed in Figure 3. In any event, as shown in Figure 4C, following the divergence of the ancestral cartilaginous fishes and the ancestral bony fishes, an interaction between MRAP1 and MC2R in the ancestral bony fish linage must have occurred. Initially this interaction could have been neutral (no apparent advantage for the function of either transmembrane protein). However, as mutations altered the trafficking features of MC2R and the ligand selectivity of MC2R, the pre-adaptation for MC2R and MRAP1 to form a complex at the ER rescued this GPCR that if expressed alone could not function properly. Since both teleost and tetrapod MC2Rs are dependent on the interaction with MRAP1 for functional expression, the interaction must have developed in a common ancestor to both the teleosts and the tetrapods. In this scenario, the interaction between MC2R and MRAP1 would have transitioned over time from a neutral interaction to a functionally dependent interaction with respect to MC2R functionality, and serves as an example of constructive neutral evolution (Stolzfus, 1999).

## Conclusion

The evolution of the MCRs is intertwined with the co-evolution of the ligand-encoding *POMC* gene, the accessory protein *MRAP* genes, and the inverse agonist *AGRP/ASIP* genes. The presence of five *Melanocortin Receptor* genes in the genomes of tetrapods indicates that the gene family has been shaped by two genome duplication events and one local gene duplication event. Based on these observations, the origin of this gene family may have occurred over 500 MYA prior to the emergence of jawless vertebrates. Synteny studies provide support for the conclusions that the local gene duplication involved the *MC2R* gene and the *MC5R* gene (Schiöth et al., 2003; Klovins et al., 2004a).

Studies on the functional activation of cartilaginous fish MCRs may provide some insights into the properties of the melanocortin receptor genes in the ancestral gnathostomes (Ringholm et al., 2003; Klovins et al., 2004b; Reinick et al., 2012a,b; Liang et al., 2013a). Current studies indicate that orthologs of MC2R, MC3R, MC4R, and MC5R can all be stimulated by ACTH or MSH-sized ligands with varying degrees of efficacy, and none of these receptors apparently requires interaction with an accessory protein to facilitate trafficking to the plasma membrane or activation once at the plasma membrane following a ligand binding event.

Among the descendents of the ancestral bony vertebrates (e.g., teleost and tetrapods) MC1R, MC3R, MC4R, and MC5R have retained the proclivity for stimulation by ACTH or the MSH-sized ligands, and none of these receptors requires an interaction with an accessory protein to facilitate trafficking to the plasma membrane. The exception to this generalization is MC2R. These features evolved in this receptor which made the receptor exclusively selective for ACTH, but also dependent on MRAP1 not only for trafficking to the plasma membrane but also for functional activation following an ACTH binding event.

The interaction between MC2R/MRAP1 in teleosts and tetrapods insures the strict signaling selectivity of the hypothalamus/pituitary/adrenal (HPA) axis and the hypothalamus/pituitary/interrenal (HPI) axis. As noted in the Introduction, MCRs are also involved in integument pigmentation, appetite regulation, glucocorticoid synthesis, and exocrine gland secretion (Gantz and Fong, 2003; Cone, 2006). The role of MCRs in these physiological processes have been extensively analyzed in mammals. For non-mammalian vertebrates it is now time to rectify the pharmacology on MCRs with the physiology of these processes in non-mammalian vertebrates. As just one example, do the cartilaginous fishes have a true HPI axis if all cartilaginous fish MCRs can be activated by either ACTH or MSH-sized ligands (Liang et al., 2013b)? What role does receptor dimerization, homo-, or hetero-play in the functionality of MCRs? Is MRAP2 an evolutionary anachronism, or does this accessory protein have a role to play in some melanocortin physiological processes? Although MCRs were characterized nearly 20 years ago, there are still many questions about this gene family that are waiting to be resolved.

## Conflict of Interest Statement

The authors declare that the research was conducted in the absence of any commercial or financial relationships that could be construed as a potential conflict of interest.
